# Polymorphism in Ontogenetic Duration in *Dendrolimus* (Lepidoptera: Lasiocampidae): Diapause, Developmental Variability, and the Role of Facultative Summer Diapause in the Siberian Moth

**DOI:** 10.3390/insects17070738

**Published:** 2026-07-19

**Authors:** Natalya A. Zhevnova, Vladimir V. Dubatolov

**Affiliations:** 1 Scientific Center of Genetics and Life Sciences, Sirius University of Science and Technology, Olympic Avenue 1, Sirius 354340, Krasnodar Krai, Russia; 2 Federal State Budgetary Scientific Institution «Federal Research Center of Biological Plant Protection», Kalinina Str, 62, Krasnodar 350039, Krasnodar Krai, Russia; 3 Federal State Institution «Zapovednoe Priamurye» Serysheva Str. 60, Khabarovsk 680038, Khabarovskii Krai, Russia; vvdubat@mail.ru; 4 Institute of Systematics and Ecology of Animals, Siberian Branch, Russian Academy of Sciences, Frunze Str. 11, Novosibirsk 630091, Novosibirsk Oblast, Russia

**Keywords:** *Dendrolimus*, Siberian moth, ontogenetic duration, intrapopulation polymorphism, developmental trajectories, diapause, facultative summer diapause, voltinism, climate adaptation, outbreak prediction

## Abstract

Representatives of the genus *Dendrolimus* are major conifer defoliators whose variable developmental duration complicates phenological forecasting, outbreak prediction, and pest-management timing. This review synthesizes evidence on polymorphism in ontogenetic duration in *Dendrolimus* and frames it at three levels: intrapopulation, interspecific, and spatial–geographical. We distinguish biological generation time, the interval from oviposition to adult emergence, from calendar span, the number of calendar years occupied by one generation; this distinction is essential for interpreting two- and three-calendar-year cycles. The evidence indicates that diapause is the main physiological mechanism generating alternative developmental trajectories, but its expression is shaped by interacting photoperiodic, thermal, trophic, density-dependent, and physiological factors. The strongest evidence for natural intrapopulation splitting is available for the Siberian moth in which fast and delayed trajectories appear associated with facultative summer diapause, or a diapause-like state. Key gaps remain concerning the induction cues, physiological status, molecular regulation, and field frequency of this state. We argue that models should incorporate delayed fractions, fast/delayed cohort ratios, and adult-emergence overlap to improve monitoring, forecasting, and control of *Dendrolimus* pests, especially under climate warming and during the early stages of outbreaks.

## Simple Summary

Representatives of the genus *Dendrolimus* are serious pests of coniferous forests. Forecasting their outbreaks is difficult because individuals do not always develop at the same speed. In some species, development may take one year in warm regions but two or more years in colder regions; in others, individuals from the same population may follow different developmental paths. This review explains how such differences arise and why they matter for forest protection. We focus on polymorphism in ontogenetic duration, that is, the coexistence of alternative developmental trajectories that differ in the time needed to complete development. Diapause, a temporary arrest of development, is the main mechanism involved. The Siberian moth is especially important because some larvae appear to enter facultative summer diapause, a poorly studied state that can delay development and complicate forecasting. Recognizing this hidden variability may improve phenological models, outbreak prediction, and the timing of monitoring and control measures.

## 1. Introduction

The genus *Dendrolimus* (*Lepidoptera: Lasiocampidae*) is generally considered to comprise 28–35 phytophagous species. Most representatives of the genus are primarily associated with coniferous trees and are distributed mainly within the Palaearctic region. Members of the genus occur across most of Europe, including several parts of Scandinavia. They are also distributed in northwestern Africa; Russia; Kazakhstan; Mongolia; China, including Taiwan; the Korean Peninsula; and Japan. Within Russia, the range of the genus extends southwards to the Caucasus, being inclusive of this region, and eastwards to Sakhalin and the southern Kuril Islands.

For some *Dendrolimus* taxa, the exact boundaries of their ranges and their taxonomic status remain disputed. The status of several species and subspecies is still being revised [1,2,3,4]. The most illustrative example is provided by two phenotypically similar groups within the genus *Dendrolimus*: *D. sibiricus* and *D. superans*. The ranges of these taxa occupy a substantial part of the Palaearctic region. According to one group of authors, including a co-author of the present review, *D. superans sibiricus* is treated as a subspecies within *D. superans superans* [5]. Other authors rely more heavily on molecular markers. However, not all such markers may be equally informative for diagnostic purposes. On this basis, they separate the taxa under consideration into two species: *D. sibiricus* and *D. superans* [3]. The divergence of these taxa was most likely relatively recent. This process may not yet be complete. However, resolving the taxonomic status of representatives of the genus *Dendrolimus* is beyond the main scope of this review. Therefore, to avoid confusion between species and subspecies names, we use the original taxon names given in the respective studies cited.

This ambiguity is important for the present review because data on life-cycle duration, diapause expression, host associations, geographical distribution, and outbreak dynamics have been published by different authors under different taxon names. This complicates cross-comparison of studies, because differences in developmental duration may reflect not only biological differences between taxa but also geographical variation, local population-specific traits, or differences in the taxonomic concept adopted by the authors. This issue is especially relevant when comparing data on the Siberian moth from Siberia, the Russian Far East, northeastern China, Sakhalin, and Japan. For this reason, we retain the original names used in the cited sources and interpret developmental and phenological data within the corresponding taxonomic and geographical context.

**Figure 1:**
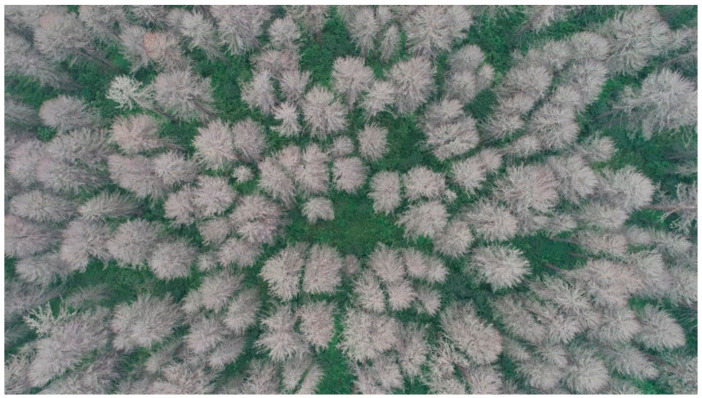
Forest stand damage in an outbreak area of Siberian Moth. Photograph courtesy of Ivan Kerchev, used with permission.

Each species is characterized by its own geographical range and host-plant spectrum. The Siberian moth, referred to in the literature as *D. sibiricus* or *D. superans sibiricus*, occupies much of Siberia and the Russian Far East. Its range also includes adjacent regions of Kazakhstan, Mongolia, and northern China [1,4,6,7,8,9,10]. The principal host plants of the Siberian moth include *Pinus sibirica*, *P. koraiensis*, *Larix sibirica*, *L. gmelinii*, *Abies sibirica*, *A. nephrolepis*, and *A. sachalinensis* [9,10,11]. The most severe outbreaks in Siberia are usually associated with stands dominated by *L. sibirica*, *A. sibirica*, and *P. sibirica* [10,12]. *Picea obovata* and *P. jezoensis* have also been reported as host plants, but they appear to represent suitable or locally important hosts rather than principal hosts [11,13]. This pest is included in the EPPO A2 List of pests recommended for regulation as quarantine pests [1]. Severe stand damage caused by Siberian moth outbreaks is illustrated in Figure 1.

The range of *D. superans* includes Japan, northeastern China, and the Russian Far East, including Sakhalin and the Kuril Islands. This species is considered an important defoliator of coniferous forests in Japan and northeastern China. Its main host plants include *Larix gmelinii*, *Pinus pumila*, *Picea jezoensis*, and *Abies sachalinensis*. Additional host plants reported for this species include *Pinus densiflora*, *P. koraiensis*, *P. thunbergii*, and *Tsuga sieboldii* [8,14,15,16,17,18,19]. The description of the range of *D. superans* depends on the taxonomic concept adopted. If *D. sibiricus* and *D. superans* are treated as separate species, their ranges are described separately. Under the subspecies concept, however, the range of *D. superans* includes the entire range of *D. sibiricus.*

The range of *D. pini* extends from Western Europe and northwestern Africa to Central Asia. It also includes China and the Asian part of Russia; the species has also been recorded in Scotland. The main host plant of *D. pini* is *Pinus sylvestris* [20,21,22]. In the absence of the main host plant, or under conditions of high population density, larvae may also feed on other tree species. These include other *Pinus* species, as well as representatives of the genera *Picea*, *Larix*, *Abies*, *Tsuga*, and *Pseudotsuga* [20]. On Kunashir Island, located in Sakhalin Oblast of the Russian Federation within the Kuril archipelago, *Pinus pumila* has been recorded as a host plant of this taxon (personal observations).

The range of *D. punctatus* includes China, including Taiwan, and Vietnam. This species has also been recorded in India and on Japanese islands located near Taiwan. *D. punctatus* damages several *Pinus* species, including *P. massoniana*, *P. merkusii*, *P. luchuensis*, and *P. tabulaeformis* [23,24,25,26].

*D. spectabilis* is an active forest pest in East Asian countries. Its range includes China, Japan, and Korea. The main host trees of this species are considered to be *Pinus densiflora*, *P. thunbergii* and *P. strobus* [2,14,27,28]. *D. tabulaeformis* is distributed in several northern Chinese provinces. This species damages several pine species, including *Pinus tabulaeformis*, *P. sylvestris var. mongolica*, *P. armandii*, *P. massoniana*, and *P. bungeana* [29,30,31,32].

In southern China, *D. houi* and *D. kikuchii* are regarded as economically important pests. For *D. houi*, the reported host plants include *P. yunnanensis*, *Cryptomeria fortunei*, and *Cupressus funebris. D. kikuchii* damages *P. langhianensis*, *P. yunnanensis*, *P. massoniana*, *P. armandii*, and *Keteleeria evelyniana* [33,34].

Representatives of the genus *Dendrolimus* are economically important defoliators of coniferous forests. Their population outbreaks are large-scale, quasi-cyclic, and stage-structured events. Studies on *D. sibiricus* have shown that a population outbreak represents a gradation process. This process includes several successive phases: a latent phase of population build-up, an increase phase, an eruptive phase of mass defoliation, and a subsequent decline. The complete cycle of outbreak development and collapse may last approximately 10 years. At the beginning of an outbreak, outbreak foci may cover only a few hectares. As the outbreak develops, their area may expand from hundreds of thousands to several million hectares. For *D. sibiricus*, historical outbreaks covering 7–8 million ha have been reported in Siberia [1,10,11]. Population density also changes sharply during the gradation cycle. At early stages, the average density may remain below one caterpillar per tree, whereas values of up to 20,000 caterpillars per tree have been reported during the eruptive phase. For *D. sibiricus*, it has been emphasized that control measures and detailed monitoring should begin 1–2 years before the population reaches its peak density. This is because, by the time the peak is reached, the outbreak has already entered the phase of mass defoliation, and the outbreak foci have already expanded substantially. Therefore, early predictors of outbreak onset and methods for the advance detection of weakened stands are of particular importance. Large-scale outbreak foci have been described for *D. sibiricus*, *D. pini*, and *D. superans* [1,6,12,20,22,35,36,37].

Aerial application of chemical insecticides remains the main form of forest protection against defoliating insects [20,38]. However, the use of synthetic chemical pesticides has become increasingly restricted in recent years. At the same time, integrated pest management (IPM) has gained importance. IPM includes preventive measures, monitoring, and control actions. Within this framework, priority is given to non-chemical control methods [25,36]. This approach can reduce pest abundance while exerting a lower impact on the forest ecosystem. Biological control is based primarily on the use of biopesticides derived from *Bacillus thuringiensis* (Bt) [20,36,39]. In addition, pheromone monitoring and pheromone-based control methods are used or being developed within pest management systems [30,37]. Control methods based on microsporidia, entomopathogenic fungi, viruses, and parasitoid insects are also under development [20,36,39].

The management of *Dendrolimus* population outbreaks requires regular monitoring, early detection of outbreak foci, and advance response. These measures are particularly important before the population enters the phase of mass defoliation [1,35,36,38,39].

Forecasting pest abundance and the risk of forest damage is based on the combination of several groups of predictors. These predictors include meteorological and hydrothermal variables, direct population indicators, pheromone-monitoring data, information on associated species, and satellite-derived metrics of forest stand condition [17,20,35,37]. However, the effectiveness of such forecasting may be limited by specific biological traits of *Dendrolimus*. These traits include cryptic larval development, a prolonged life cycle, diapause, and developmental asynchrony among individuals. These characteristics may obscure the early stages of population build-up. As a result, timely detection of the transition to outbreak dynamics becomes more difficult. For the Siberian moth, the same predictors [17,20,35,37,40,41,42,43] may also be tested as factors associated with the split between fast-developing and delayed larvae, including the probability of facultative summer diapause or an additional overwintering event [4,44,45,46].

In representatives of the genus *Dendrolimus*, life-cycle duration varies widely. In northern and temperate forms, development may extend over several years, whereas southern species may produce several generations per year [6,25,28,30,38,47,48].

Against this background, the Siberian moth occupies a particular position. This pest is typically characterized by two main developmental trajectories: a two-year developmental cycle that spans three calendar years, and a one-year developmental cycle that spans two calendar years. At the same time, some reports indicate that development may be completed within one year or, in exceptional cases, may extend to 4–5 years. This variability is associated with atypical facultative summer diapause. This case apparently cannot be fully explained by the known ecological and physiological mechanisms of regulation. It therefore occupies a special position among examples of intrapopulation polymorphism in ontogenetic duration in *Lepidoptera* [1,4,6,44,45,49].

These life-cycle traits substantially complicate population-outbreak forecasting and the development of long-term strategies for managing *Dendrolimus* populations. Because some individuals may remain in diapause or delayed development for several years, control measures targeting a single generation may be less effective than expected. In this situation, monitoring based on a single expected age class may underestimate the hidden delayed fraction [4,44,45,46], whereas chemical or biological treatments timed for one vulnerable cohort may leave larvae that are physiologically out of phase with the target window [20,35,36,37,38,39].

The aim of this review is to synthesize current knowledge on variation in ontogenetic duration in conifer-feeding pests of the genus *Dendrolimus*, with particular emphasis on the Siberian moth. Specifically, this review aims to summarize the available evidence for intra- and interspecific variation in life-cycle duration within *Dendrolimus*; analyze the ecological, physiological, and genetic factors that may contribute to alternative developmental trajectories, including delayed development and facultative summer diapause; clarify the relationship between polymorphism in ontogenetic duration, diapause, voltinism variation, and outbreak dynamics; and discuss how developmental heterogeneity can be incorporated into phenological forecasting, outbreak prediction, and pest-management strategies under changing climatic conditions.

## 2. Conceptual Framework: Polymorphism in Ontogenetic Duration and Voltinism Variation

In this review, we use the term “polymorphism in ontogenetic duration” in a broader sense than “voltinism variation”. Voltinism describes the number of generations completed by a population or species within a calendar year and is therefore a phenological descriptor of annual generational output [50,51,52]. By contrast, polymorphism in ontogenetic duration refers both to variation in the time required to complete ontogenesis from egg to adult and to the coexistence or differentiation of alternative developmental trajectories. These trajectories may differ in growth rate, number of overwintering events, diapause expression, diapause duration, and timing of development resumption [51,52,53,54,55].

**Figure 2:**
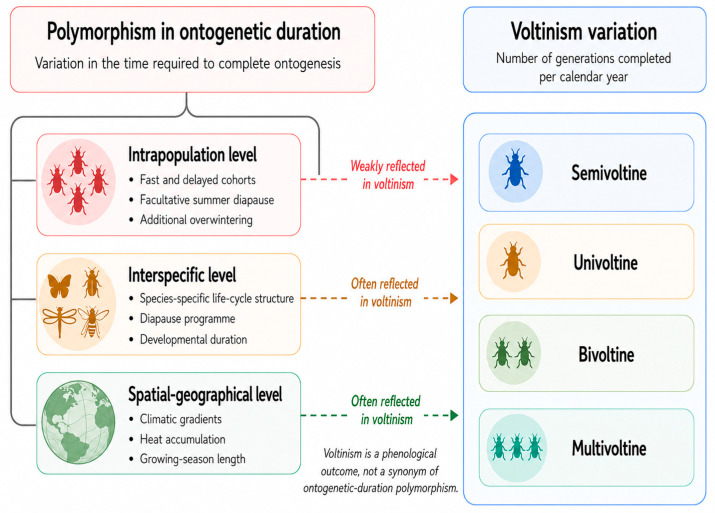
Conceptual relationship between polymorphism in ontogenetic duration and voltinism variation.

In *Dendrolimus*, this distinction is important because variation in ontogenetic duration is expressed at several biological levels. At the intrapopulation level, individuals or cohorts within the same population may follow fast or delayed developmental trajectories, as described for the Siberian moth [44,45]. At the interspecific level, different *Dendrolimus* species differ in the typical organization of the life cycle, diapause expression, developmental duration, and voltinism [20,24,28,29,32,38,48,56]. At the spatial–geographical level, populations of the same species may differ in developmental duration along climatic gradients, reflecting differences in heat accumulation, growing-season length, and seasonal constraints [6,8,10,12]. Voltinism may reflect some of these differences, especially at the interspecific and geographical levels, but it does not by itself describe the internal structure of alternative developmental trajectories. Therefore, in this review, voltinism is treated as one possible phenological outcome of ontogenetic-duration variation, rather than as a synonym for it. This conceptual relationship is summarized in Figure 2.

Polymorphism in ontogenetic duration in representatives of the genus *Dendrolimus* can be distinguished at least at three levels. The first is intrapopulation variation, which occurs when individuals with alternative developmental trajectories coexist within a single local population. The second is interspecific variation, reflecting differences among *Dendrolimus* species in the typical structure of the life cycle. The third is geographical variation, expressed as differences among populations of the same species in different parts of the range. At this level, developmental duration and voltinism may vary with climate, growing-season length, and other regional factors.

**Figure 3:**
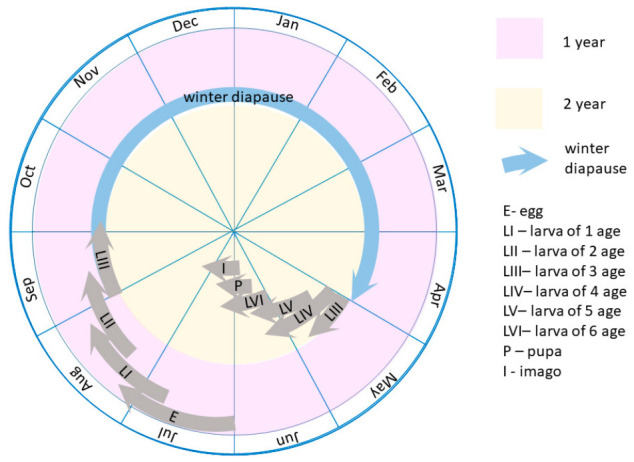
Phenological scheme of the two-calendar-year developmental cycle of the Siberian moth, based on Baranchikov et al. (1997) [46]. Oviposition occurs from late June to mid-July. Larvae develop during the first growing season, overwinter in the litter, resume feeding in spring of the following calendar year, and pupate in June or early July. Larval instars are indicated as L1–L6. The shaded winter period indicates larval overwintering associated with winter diapause. Background colors indicate the calendar-year zones and diapause periods, as shown in the legend.

**Figure 4:**
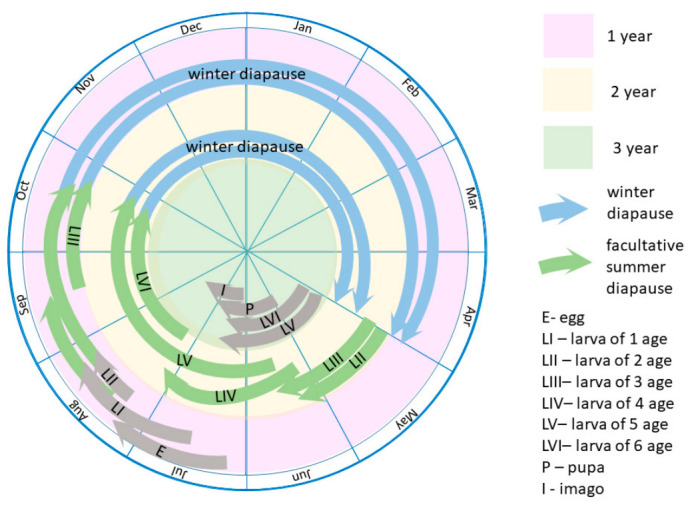
Phenological scheme of the three-calendar-year developmental cycle of the Siberian moth, based on Baranchikov et al. (1997) [46]. Oviposition occurs from late June to mid-July. Larvae develop during the first growing season and overwinter in the litter. In the second calendar year, larvae resume feeding but do not complete development; instead, development is prolonged through facultative summer diapause, followed by an additional overwintering event. Pupation occurs in the third calendar year. Larval instars are indicated as L1–L6. Shaded periods indicate winter diapause/overwintering and facultative summer diapause. Background colors indicate the calendar-year zones and diapause periods, as shown in the legend.

In representatives of the genus *Dendrolimus*, intrapopulation differentiation in developmental rate has been most clearly demonstrated in the Siberian moth (*D. sibiricus* or *D. superans sibiricus*). In this pest, a condition referred to as facultative summer diapause has been described: at a certain developmental stage, only a proportion of larvae in the population enter diapause, whereas others continue development without diapause. This state may occur under both favorable and unfavorable environmental conditions. As a result, individuals following fast and delayed developmental trajectories coexist within the same population during the same time period. Thus, the population is effectively divided into two groups: individuals whose life cycle lasts 2 calendar years (Figure 3), and individuals whose life cycle lasts 3 calendar years (Figure 4) [44,45,57].

Development according to the classical two-year cycle, which spans two calendar years, begins with oviposition from late June to mid-July. During the first season, larvae develop to the third or fourth instar and then overwinter in the litter. In spring of the following calendar year, they emerge from their overwintering sites, feed intensively, and, after completing development, pupate in June or early July of the same year [4,6,23,45] (Figure 3).

Development according to the cycle spanning three calendar years also begins with oviposition from late June to mid-July. During the first season, larvae develop to the second or third instar and then enter overwintering. In spring of the following calendar year, larvae emerge from their overwintering sites but do not complete development. Despite favorable conditions for further growth, they enter facultative summer diapause. The larvae then continue development to the fourth or fifth instar and overwinter again. Pupation occurs in the third calendar year [4,23,44,45] (Figure 4).

The extreme variants of life-cycle duration in the Siberian moth (*D. sibiricus* or *D. superans sibiricus*)—the one-year and 4- to 5-year variants—have been described only fragmentarily in the literature [4,8,10,45]. Both variants should be considered primarily at the geographical level of intraspecific variability. One-year development is usually associated with the southern parts of the natural range and high heat availability. According to EFSA, it is possible when the sum of degree-days above 10 °C exceeds 2200 [6,10]. EPPO also indicates that, in the southern part of the range, one generation may be completed within one year [8]. By contrast, prolonged development to 3–4 years, and in exceptional cases to 5 years, has been reported for northern parts of the range [4,8,45]. Therefore, both the one-year and the 4- to 5-year variants should be regarded primarily as extreme manifestations of geographical variation in seasonal development. This variation is likely determined by a combination of climatic conditions and population-specific traits.

However, the evidential status of these extreme variants differs from that of the two- and three-calendar-year cycles described for natural populations. The one-year and 4–5-year variants are supported mainly by scattered field observations, pest reports, and range-level summaries rather than by detailed experimental or long-term cohort studies. In other words, these records are interpreted here as examples of geographic intraspecific variation, whereas incidental within-population variation would require direct evidence that such extreme trajectories occur simultaneously within the same local population. Therefore, in this review they are treated as rare or geographically constrained manifestations of life-cycle plasticity, not as well-characterized intrapopulation developmental trajectories. For forecasting purposes, these extreme variants should therefore be used cautiously. They indicate the potential range of developmental plasticity in the Siberian moth, but they do not yet allow reliable estimation of the frequency, environmental thresholds, or population-level contribution of such trajectories in natural populations.

When describing the life cycle of *D. sibiricus*, it is important to distinguish between two concepts. The first is the biological duration of a generation, that is, the period from oviposition to adult emergence. The second is the calendar span of a generation, that is, the number of calendar years encompassed by the development of a single generation when oviposition occurs in summer of one year and adult emergence occurs in summer of the following or a subsequent year. It is likely that, in descriptions of the life cycle of this pest, different authors have not always consistently distinguished between these concepts.

Partial differentiation of a population into diapausing and non-diapausing fractions has been demonstrated in a Chinese population of *D. punctatus*. However, this differentiation was observed under strictly controlled laboratory conditions. Within a single *D. punctatus* population, exposure to the critical photoperiod of 13.5:10.5 L:D resulted not in complete induction but in partial, diapause induction. The proportion of diapausing larvae was 51.7%, 70.8%, and 81%, depending on the degree of damage to the pine needles used as the food source. Under long-day conditions, diapause was completely absent, whereas under short-day conditions, 100% of larvae entered diapause. This indicates that, near the critical photoperiodic threshold, individuals from the same population split into development-continuing and diapausing fractions [58].

The pattern described in *D. pini* has a different nature. In this species, variation in life-cycle duration from 1 to 3 years is associated with climatic conditions and the thermal regime. This case should therefore be regarded primarily as geographical intraspecific variability. In different parts of its range, the same species realizes life cycles of different durations [20,38].

A similar example of geographical intraspecific variability has been described in *D. spectabilis*. In central Korea, a local population was shown to shift from univoltinism to bivoltinism. This shift has been associated with increased heat accumulation and regional climatic change [56].

Thus, polymorphism in ontogenetic duration in representatives of the genus *Dendrolimus* has a multilevel structure. Distinguishing among intrapopulation, geographical, and interspecific levels helps identify the regulatory and ecological processes that generate particular life-cycle structures. This distinction is also important for accurate phenological forecasting.

To make these relationships explicit, Table 1 summarizes the main levels of ontogenetic-duration polymorphism documented or inferred across selected *Dendrolimus* taxa.

## 3. The Role of Diapause in Shaping Polymorphism in Ontogenetic Duration

Diapause is the central physiological mechanism through which differences in ontogenetic duration are realized. It is a complex, physiologically mediated dynamic process. This process consists of several successive phases and occurs at a specific developmental stage. Diapause synchronizes the life cycle with seasonally recurring adverse environmental conditions. It may develop as an anticipatory adaptive program induced by seasonal cues. In other cases, it may be fixed in the life cycle as an obligate stage [50,53,54,67,68].

Diapause is one of the most important mechanisms by which insects adapt to seasonal environmental variation. It increases resistance to cold, heat, desiccation, and food shortage [55]. The incidence of diapause may vary substantially not only among species but also among different populations of the same species [68].

Diapause is commonly regarded as a process consisting of several successive phases [54]:

- Pre-diapause is the phase of physiological and behavioural preparation for the subsequent arrest of development. It may be initiated before the onset of adverse environmental conditions. In some cases, this phase may be activated without the direct involvement of external cues.
- Diapause is an endogenously regulated arrest of development. At this stage, the organism follows an alternative physiological program that may be modulated by environmental conditions. In some organisms, feeding, accumulation of energy reserves, and searching for a suitable habitat may continue during this stage.
- Post-diapause corresponds to the phase of diapause termination. At this stage, the resumption of direct development becomes physiologically possible. However, it may not occur until favorable environmental conditions are restored.

Each phase of diapause may include several subphases. Their expression is determined by both genotype-dependent physiological changes and environmental conditions [53,54].

The onset of diapause usually anticipates the beginning of an adverse period. However, diapause termination does not necessarily coincide with the end of this period. Therefore, two parameters are particularly important for the formation of polymorphism in ontogenetic duration: diapause intensity and the post-diapause stage. Diapause intensity determines the duration of the dormant state. The post-diapause stage, in turn, determines when development can resume depending on environmental conditions. As a result, even when the duration of diapause itself is similar, individuals may complete development at different times. This leads to developmental asynchrony and the formation of different developmental trajectories [55].

Diapause can be classified according to several criteria [51,53,54,68]:

- Based on developmental stage, embryonic, larval, pupal, and adult, diapause can be distinguished [51,53,68].
- Based on the mode of induction, diapause can be either obligate or facultative. Obligate diapause is genetically fixed and occurs in all individuals of a generation. Facultative diapause is induced in response to environmental cues and may not be expressed in all individuals of a population [51,53,54].
- Based on seasonal timing, winter and facultative summer diapause can be distinguished [50,54].
- Based on duration, diapause can be classified as normal or prolonged [51,52,68].

This classification is important for understanding how ontogenetic-duration polymorphism is expressed at different hierarchical levels [51,53,54]. Winter diapause represents the basic seasonal mechanism that allows larvae to survive unfavorable cold periods and structures the annual rhythm of development. In contrast, facultative summer diapause may act as a branching point within the population. Individuals that avoid this state can continue along a faster trajectory. Individuals that enter it shift to a delayed trajectory and may require an additional season or overwintering event to complete development [4,6,8,9,18,44,45]. Repeated overwintering and prolonged larval development further extend this delay and contribute to multi-year life cycles [4,6,8,9,18,44,45,53,54]. At the intrapopulation level, different combinations of these diapause states generate fast- and delayed-developing cohorts within the same population. At the interspecific level, species-specific differences in the occurrence, timing, and intensity of winter and facultative summer diapause contribute to differences in typical life-cycle duration among *Dendrolimus* species [28,38,48,51,53,54]. At the geographical level, climatic seasonality, heat accumulation, photoperiodic background, and host-plant conditions may change the relative frequency of these diapause combinations, thereby modifying the proportion of short and prolonged developmental cycles across the range [53,54,69,70].

At the intrapopulation level, variation in diapause expression among individuals within the same population is of key importance. Polymorphism in ontogenetic duration may arise in several ways: when diapause is induced only in some individuals; when it differs in intensity, duration, or timing of termination; or when it becomes prolonged in a fraction of individuals. As a result, alternative developmental trajectories differing in life-cycle duration emerge within a single population [53,54,68,71,72,73,74,75,76].

At the interspecific level, diapause represents one of the traits defining the overall organization of the life cycle. Species may differ in the developmental stage at which diapause occurs, in its seasonal timing, in the degree to which it is obligate or facultative, and in the tendency toward prolonged development. These differences determine the degree of life-cycle plasticity and may explain interspecific variation in phenology, voltinism, and ontogenetic duration [51,53,54,77].

At the spatial–geographical level, variation in diapause is manifested primarily as differences among populations of the same species inhabiting different climatic conditions. These differences may involve photoperiodic and temperature thresholds for diapause induction and termination, as well as diapause intensity, duration, and the frequency of prolonged development. As a result, spatial differences in phenology, voltinism, and life-cycle duration arise within the species range [53,54,69,70].

In representatives of the genus *Dendrolimus*, diapause is associated with life-cycle duration at several levels: intrapopulation, interspecific, and spatial–geographical. It represents an important component of the seasonal organization of the life cycle and may substantially affect its duration. This relationship is expressed differently among representatives of the genus. In *D. punctatus*, larval diapause is associated with differences between univoltine and multivoltine populations. In *D. spectabilis*, winter diapause forms part of the seasonal cycle and may be associated with changes in voltinism under warming conditions. In *D. pini*, differences in the number of overwintering events accompany the transition from one-year to two- and three-year development under cooler conditions. At the spatial–geographical level, regional variation may occur in the thresholds of diapause induction, as well as in diapause intensity, duration, and frequency of expression. These changes, in turn, may lead to differences in life-cycle duration [28,38,48].

Intrapopulation variability in life-cycle duration has been described in *D. sibiricus*, a pest treated in some taxonomic studies as *D. superans sibiricus*. In this case, intrapopulation polymorphism in ontogenetic duration is associated with a combination of winter diapause and facultative summer diapause. This combination may prolong the life cycle from 2 to 3 calendar years and, according to some sources, up to 4–5 calendar years [4,6,8,9,18,44,45].

The presence of facultative summer diapause in *D. sibiricus* results in the simultaneous occurrence of individuals or cohorts with different developmental durations within the same population. Some individuals follow a two-year developmental cycle (Figure 3), whereas others follow a three-year developmental cycle (Figure 4).

Similar examples of intrapopulation splitting of developmental trajectories through variable diapause expression are also known in other Lepidoptera. In *Choristoneura fumiferana*, different developmental rates may occur within a single population. Most individuals follow a one-year life cycle; however, some individuals either do not enter diapause or, conversely, undergo an additional diapause. In the latter case, development may be prolonged to two years [78].

In *Zygaena trifolii*, alternative developmental trajectories may also occur within a single population. Even under identical conditions, larvae may enter diapause at different instars, and some individuals may undergo repeated diapause. As a result, fast- and delayed-developing individuals are formed within the population, and the overall duration of the life cycle may vary from one to 3–4 years [79].

In *Thaumetopoea pityocampa*, intrapopulation splitting of developmental trajectories has also been demonstrated. Among individuals collected from the same site in the same year, individuals with direct adult emergence and individuals with prolonged pupal diapause were present simultaneously. In the latter group, diapause could last for one or several additional years [80].

Thus, intrapopulation variability in ontogenetic duration in *D. sibiricus* can be considered a specific case of a more general mechanism in *Lepidoptera*. Alternative developmental trajectories are formed not by replacing the seasonal program itself but by differences in diapause expression: which individuals enter diapause, at which developmental stage, with what intensity, and for how long.

This comparison indicates that facultative or prolonged developmental delay is not unique to *Dendrolimus* in a broad sense, because alternative diapause-mediated trajectories also occur in *Choristoneura fumiferana*, *Zygaena trifolii*, *Thaumetopoea pityocampa*, and other insects [78,79,80,81]. The specificity of the Siberian moth lies rather in the apparent coupling of larval facultative summer diapause with natural intrapopulation splitting into fast and delayed trajectories and with two- and three-calendar-year population structure [4,44,45,46,59,60,61,62,63]. In the non-*Dendrolimus* examples discussed above, developmental divergence is described mainly through additional diapause, repeated larval diapause, or prolonged pupal diapause, usually within a framework of risk spreading, threshold responses, or bet-hedging [72,74,78,79,80,81]. For *D. sibiricus*, by contrast, the available data suggest that the relevant switch cannot yet be reduced to classical short-day induction of winter diapause. It is more likely to depend on a combination of thermal conditions, trophic stress, population density, larval physiological state, and developmental history [45,59,60,61,62,63,64]. Therefore, facultative summer diapause in the Siberian moth should currently be treated as a mechanistically unresolved but biologically distinct form of developmental delay, and strict mechanism-by-mechanism comparison with other *Lepidoptera* remains premature until direct experimental and molecular data become available [64].

## 4. The Evolutionary Basis of Polymorphism in Ontogenetic Duration

In representatives of the genus *Dendrolimus*, life-cycle duration is not a strictly fixed species-specific trait. It may vary depending on climatic conditions, the geographical position of the population, the length of the growing season, trophic conditions, and the physiological state of larvae. This feature is particularly pronounced in *D. sibiricus*, for which variation in life-cycle duration has been described, including both typical and prolonged developmental trajectories.

The ability of insects to vary ontogenetic duration within a single population is regarded as an important adaptation to seasonal and interannual environmental variability. This polymorphism is manifested in the coexistence of individuals that complete development over different numbers of seasons. It is often mediated by variation in diapause, including its prolongation for one or several additional cycles [53,54].

According to evolutionary models, the distribution of diapause termination among different years corresponds to a bet-hedging strategy. This strategy reduces the risk of complete loss of a generation during unfavorable periods [72,74,81]. Mathematical models also show that, in stochastically varying environments, mixed strategies of diapause termination may provide higher long-term fitness than fixed strategies.

The evolutionary significance of variation in diapause duration becomes particularly important under high interannual climatic variability. Under such conditions, favorable periods for insect development and reproduction occur irregularly. When diapause termination is distributed across several seasons, the population reduces the risk that the entire life cycle will become synchronized with adverse environmental conditions [72,74,75,81,82].

Theoretical studies also show that an increase in the maximum duration of diapause may substantially increase the probability of population persistence. This effect is especially important in positively autocorrelated environments, where adverse periods may last for several consecutive years [83]. Under such conditions, prolonged diapause may function as a mechanism of population “insurance” against extended adverse environmental phases.

In the context of *Dendrolimus*, this evolutionary logic should be considered at several levels. At the intrapopulation level, it is manifested in the coexistence of individuals with different developmental durations within a single local population. At the spatial–geographical level, the frequency of fast and delayed developmental trajectories may vary along climatic gradients. Such variation may reflect differences in the length of the growing season, the sum of effective temperatures, and the predictability of seasonal conditions. At the interspecific level, closely related *Dendrolimus* species may differ in the typical structure of the life cycle and in the expression of diapause. These differences can be interpreted as different evolutionary solutions to the common problem of seasonal synchronization of development.

General patterns of risk spreading described in other insects can help interpret this phenomenon in *Dendrolimus*. In the chestnut weevil *Curculio elephas*, some individuals delay development and emerge from the soil after two or three years instead of one. This distribution of adult emergence across several seasons reduces the risk that all individuals will emerge simultaneously in an unfavorable year and corresponds to a strategy of stochastic plasticity [75,84,85].

Experiments on *Zygaena trifolii* have also shown that larval diapause may have a polygenic threshold basis and may be maintained as an intrapopulation adaptive polymorphism [82]. These examples do not replace data on *Dendrolimus*, but they provide a theoretical framework for interpreting variation in developmental duration as the result of interactions among environmental cues, physiological state, and heritable thresholds of seasonal development.

## 5. Ecological Drivers of Polymorphism in Developmental Duration

The influence of environmental conditions on polymorphism in ontogenetic duration can be considered at two levels.

- Induction of differences in individual development. Ecological factors, including photoperiod, temperature, and food quality, may affect the growth rate of individual organisms and the timing of diapause induction. As a result, some individuals complete development within the current season, whereas others enter diapause or delay development. This leads to the formation of cohorts with different life-cycle durations [53,54,86].
- Population-level maintenance of polymorphism in ontogenetic duration. Under interannual environmental instability, natural selection may maintain the coexistence of individuals with different developmental durations. Distributing diapause termination or completion of ontogenesis across several seasons reduces the risk of complete synchronization with adverse conditions. In this context, variation in developmental duration can be regarded as a form of risk spreading, or bet-hedging, that increases population persistence over time [74,81,87].

The ecological factors most directly implicated in the regulation of diapause and life-cycle duration in *Dendrolimus* include photoperiod, temperature regime, and trophic conditions, including host-plant quality and food availability [24,29,32,48,58,64,65,66]. Population density may contribute indirectly by altering food availability, larval competition, and the physiological state of larvae; however, experimental data for *D. sibiricus* indicate that high larval density alone does not trigger prolonged development [64].

In the context of polymorphism in ontogenetic duration, ecological factors should be considered not as isolated causes but as a system of cues that determine the probability that alternative developmental trajectories will be realized. The same factor may operate at different levels. At the intrapopulation level, it may influence the splitting of individuals by growth rate, timing of diapause, and number of overwintering events. At the interspecific level, it may be expressed through differences among species in photoperiodic and temperature thresholds. At the spatial–geographical level, it may act through variation in growing-season length, the sum of effective temperatures, and the structure of seasonal constraints.

Therefore, photoperiod, temperature, trophic conditions, and population density should be regarded as ecological determinants. They provide the external context in which internal physiological and genetic mechanisms of seasonal development are expressed [51,53,54,69,70,73].

Photoperiod is regarded as one of the main ecological triggers of diapause in insects. It is perceived by the circadian system, which measures night length. During the photoinducible phase, photoperiod may determine the switch between direct development and diapause development [53,70]. In general, short-day conditions induce diapause, whereas long-day conditions promote the continuation of direct development [53].

However, photoperiod should not be considered the only factor inducing diapause. In Lepidoptera, diapause induction may also be determined by other environmental cues. In some cases, temperature may play a decisive role and can completely override the photoperiodic response [88]. Diapause induction may also be associated with deteriorating trophic conditions [89,90]. In addition, further regulation may occur through maternal effects [91]. Therefore, diapause induction may be determined not by a single cue but by a combination of ecological and physiological factors.

Near critical photoperiods, photoperiod should be viewed not as a fixed developmental command but as a threshold background against which other cues modify the probability of diapause induction. Under such conditions, temperature may amplify diapause incidence by slowing larval growth and reducing heat accumulation, thereby increasing the probability that larvae reach the developmental decision point in a physiological state favoring diapause; conversely, warmer conditions may weaken this response [51,53,54,88]. Host-plant quality can act in a similar way. In *D. punctatus*, the deterioration of pine-needle quality under the critical photoperiod of 13.5:10.5 L:D increased diapause incidence from 51.7% to 70.8–81.0%, while also slowing development to the third instar and reducing amino-acid and sugar contents but increasing tannin levels in the needles [58]. Food stress may also interact with larval density not as an independent crowding trigger alone but through intensified competition, faster depletion of preferred tissues, and deterioration of the remaining food, which together reduce body-mass gain and may increase the probability of delayed development [51,73,92,93]. For the Siberian moth, however, currently available experiments indicate that high density, starvation, or host-plant species, when tested separately, do not independently trigger prolonged development [64]. This suggests that in *D. sibiricus* these factors are more likely to act jointly, sequentially, or in a context-dependent manner against a near-threshold photoperiodic and thermal background.

Different representatives of the genus *Dendrolimus* show common principles of the photoperiodic regulation of the life cycle. In general, winter diapause is induced by short-day conditions. However, other factors, including temperature and food quality, may modify this process.

In *D. pini*, larval diapause is not strictly fixed to a single instar: short-day exposure beginning at the first instar induced diapause in the third instar, whereas exposure beginning at the third instar induced diapause in the fourth instar [65]. In *D. tabulaeformis*, a short-day regime of 8L:16D was used to induce diapause, whereas a long-day regime of 16L:8D was used to maintain non-diapause development [66]. In *D. punctatus*, photoperiod is regarded as the main factor inducing diapause. However, temperature [24] and host-plant quality [58] act as modifying factors. Their effects are most pronounced near critical photoperiodic values.

A different situation has been described for the Siberian moth. In this pest, facultative summer diapause leads to the splitting of the population into fast- and delayed-developing individuals and may occur even under long-day conditions. In the laboratory study by N. Kirichenko et al. [45], *D. sibiricus* larvae were reared under an L:D 18:6 photoperiod. Despite these conditions, some individuals did not complete development by the end of the experiment. This suggests that prolonged development may occur outside direct dependence on classical photoperiodic regulation. Earlier studies also indicate that facultative summer diapause in the Siberian moth may be associated with trophic stress, starvation, and temperature anomalies. Baranchikov et al. [59] demonstrated the role of starvation in the occurrence of facultative summer diapause in natural populations. Geispits [60] discussed the role of photoperiodic and temperature reactions, including abnormal temperatures, in the seasonal development of *D. pini* and *D. sibiricus*. Similar assumptions concerning the role of environmental and trophic factors were proposed by Konikov et al. [61,62]. However, recent experimental data by Akhanaev et al. [64] showed that larval density, food deprivation, and host-plant species, when tested separately, affected larval stress, mortality, and body-mass gain but did not independently trigger prolonged development. Thus, the causes of facultative summer diapause in the Siberian moth should not be regarded as completely unknown. Available data suggest the involvement of trophic and temperature-related factors. However, the switch to a prolonged developmental trajectory probably depends on a more complex combination of cues, their sequence, ecological history, and/or transgenerational effects. The evidence underlying these observations and interpretations is summarized in Table 2, where direct experimental observations, field-based evidence, historical interpretations, and model-based hypotheses are distinguished.

The role of facultative summer diapause in outbreak formation was recently emphasized by Demidko et al. [63], who considered facultative summer diapause, especially its avoidance under favorable weather conditions, as a key mechanism triggering the transition of Siberian moth populations toward outbreak dynamics. In their interpretation, weather conditions that prevent larvae from entering facultative summer diapause may accelerate larval development, promote a shift toward a one-year life cycle, and thereby contribute to rapid population increase before an outbreak.

It should also be noted that the northern boundary of the range of *D. sibiricus* or *D. superans sibiricus* reaches 63.3° N according to some sources [6,94] and 52–59° N according to others [12]. These latitudes, at least in part, fall within the zone of white nights. This may suggest that facultative summer diapause can be initiated outside direct dependence on classical photoperiodic regulation. However, the available data on northern populations do not yet allow the conditions of white nights to be directly linked to the induction of prolonged development.

In addition to photoperiod, temperature regime is a key ecological factor determining insect developmental rate and the duration of ontogenesis, because temperature directly affects metabolic intensity [50,51,52,54]. Low temperatures slow development. Under seasonal conditions, this may increase the number of overwintering events and prolong the life cycle, which is particularly important for species with larval overwintering and multi-year development, including *D. sibiricus* [1,8,52]. Temperature also regulates diapause intensity and termination, thereby determining the timing of diapause exit and the synchronization of post-diapause development [95].

The spatial–geographical level of this relationship is particularly evident in the Siberian moth. In classical descriptions of this species, life-cycle duration is linked to the thermal balance of the season. When the accumulated temperature is insufficient, development is prolonged and includes additional overwintering events. Conversely, higher heat availability increases the probability of life-cycle shortening. A normal complete developmental cycle requires an annual accumulated temperature close to 2000°. When this annual sum increases to 2100–2200°, the pest shifts to a one-year developmental cycle [6]. Recent data are consistent with this logic. In the northern part of the range, within the area of a large outbreak in the Yenisei region, a shift from a two-year to a one-year generation was recorded against the background of warming, drought, and increased accumulated temperature. The authors emphasize that this phenomenon is neither trivial nor self-evident for a northern population of this species [12].

A similar principle has also been shown for *D. spectabilis*. Model calculations based on accumulated effective temperatures predict an increase in voltinism under climate warming. These changes are expressed along altitudinal and latitudinal gradients [28].

In the review by Skrzecz et al. (2020) [20], increased air temperature, drought, and extension of the growing season are reported to potentially enhance the activity of *D. pini* in Central Europe. Over the past two decades, an expansion of the range of this species has been observed. In addition, the intervals between outbreaks have shortened from 4–16 years to 2–3 years.

Experimental studies on *D. tabulaeformis* have shown that temperature affects not only larval survival but also the successful completion of diapause. Diapausing larvae tolerate low temperatures better, and cold acclimation increases their resistance. By contrast, deacclimation and prolonged exposure to above-zero temperatures increase larval mortality [29,32].

For *D. superans sibiricus*, bioclimatic modeling has shown that one of the key limiting parameters is the sum of effective temperatures above 5 °C. This parameter determines the climatic suitability of a territory for the development of the species [57].

In addition to climatic cues, trophic conditions may affect the probability of diapause onset and its duration. Food quality and availability, as well as population density, may alter larval growth rate. In this way, they may indirectly influence the timing of diapause induction and the duration of the life cycle [51,73].

For the Siberian moth, this relationship is especially relevant because facultative summer diapause has been repeatedly discussed in connection with trophic stress, starvation, and food availability. Earlier studies by Baranchikov et al. [59] and Konikov et al. [62] considered facultative summer diapause as part of the alimentary or trophic adaptation of larvae under deteriorating feeding conditions.

Food shortage or low food quality is thought to slow larval growth and limit the accumulation of energy reserves. These reserves are required for the completion of development and the successful passage through diapause [73]. Under such conditions, individuals may fail to reach critical developmental stages within a single season. Consequently, they may enter an additional diapause, leading to the formation of cohorts with different developmental durations [51,53,54].

For *D. sibiricus*, Kirichenko et al. (2011) [45] showed, in an experiment with potted plants of different host species, that host-plant quality affects larval development. Favorable hosts (*A. grandis, L. decidua, P. menziesii*) supported higher survival and better larval development. This resulted in heavier pupae and longer-lived adults.

A more direct example of the relationship between trophic conditions and diapause regulation has been obtained for *D. punctatus*. Huang et al. (2008) [58] showed that, near the critical photoperiod of 13.5:10.5 L:D, host-plant quality markedly modified the photoperiodic response. The proportion of diapausing larvae was 51.7% on undamaged needles, 70.8% when needle damage reached 25–40%, and 81.0% when damage reached 75–90%. Needle damage also slowed larval development to the third instar: 12.0 ± 0.5, 13.3 ± 0.4, and 14.6 ± 0.7 days, respectively.

This effect was accompanied by deterioration in the chemical composition of the needles. The amino acid content decreased from 84.90 to 75.31 mg/g, and the sugar content decreased from 169.97 to 155.90 mg/g. By contrast, tannin content increased from 31.35 to 33.75 mg/g. These data indicate that trophic stress may not only reduce growth rates but also modify photoperiodic diapause induction, thereby increasing the probability that some individuals shift to a delayed developmental trajectory [58].

Population abundance may influence ontogenetic duration indirectly. This effect may be mediated by changes in food availability and the level of intraspecific competition. Review data on holometabolous insects show that larval density can substantially affect growth rate, survival, and other life-history traits. In some cases, it may also influence the probability of diapause induction [92,93]. However, the direction of this effect is not universal and depends on the biology of the species and environmental conditions.

In Siberian moth the effect of population abundance on ontogenetic duration is also likely to be mediated primarily indirectly. It may be associated with changes in food availability, increased intraspecific competition, and changes in the physiological state of larvae.

During population growth, under favorable weather and trophic conditions, some individuals may develop more rapidly. In this case, larvae may undergo only one overwintering event, which can contribute to accelerated population growth. By contrast, when food resources deteriorate, when larvae feed on secondary needles, or during periods of starvation, development may slow down. Earlier studies by Baranchikov et al. [59] and Konikov et al. [62] linked such trophic stress with facultative summer diapause and the formation of delayed developmental trajectories in the Siberian moth. Under such conditions, a three-year cycle may be formed [4,6,44]. Thus, population density itself is not necessarily a direct trigger of prolonged development. However, it may alter the ecological background against which fast or delayed developmental trajectories are realized.

However, Akhanaev et al. [64] specifically examined the contribution of ecological factors to switching of the life-cycle program in *D. sibiricus*. Under laboratory conditions, the authors tested three factors: larval density, food deprivation, and host-plant species. The authors concluded that high larval density is not an independent trigger of prolonged development. Starvation induced stress and mortality but did not result in prolonged development. A change in host plant significantly affected larval mortality and body-mass gain. However, all surviving larvae successfully pupated, and no prolonged development occurred [64].

These data suggest that switching of the developmental program in *D. sibiricus* is probably not determined by a single isolated factor. Rather, it may depend on a more complex combination of cues, their sequence, ecological history, and/or transgenerational effects. Thus, the ecological drivers of polymorphism in ontogenetic duration in *Dendrolimus* include the combined effects of photoperiod, temperature, the sum of effective temperatures, host-plant quality, population density, humidity, and overwintering conditions. Photoperiod provides a seasonal cue that determines diapause induction in photoperiodically sensitive species. Temperature and the sum of effective temperatures regulate developmental rate and the possibility of completing ontogenesis within a single season. Trophic conditions affect larval growth, survival, and energetic state. Climatic and geographical environmental heterogeneity, in turn, may maintain the coexistence of alternative developmental trajectories [12,24,28,45,50,51].

As a result, multilevel polymorphism in ontogenetic duration occurs within the genus *Dendrolimus*. At the intrapopulation level, it is expressed through the splitting of individuals into fast and delayed cohorts. At the spatial–geographical level, it is associated with differences among climatic zones. At the interspecific level, it reflects species-specific adaptation to different seasonal regimes [1,6,8,18,23,28].

## 6. Physiological Regulation of Polymorphism in Ontogenetic Duration

Polymorphism in ontogenetic duration can be considered a population-level manifestation of interindividual variability in the physiological regulation of development. This variability may affect growth rate, the probability of entering diapause, diapause intensity, the duration of developmental delay, and the timing of post-diapause growth resumption, thereby forming alternative developmental trajectories. Diapause underlies this process but does not by itself explain polymorphism in ontogenetic duration. Such polymorphism arises when the physiological regulation of diapause and growth rate varies among individuals, populations, or species. As a result, alternative developmental trajectories with different total life-cycle durations are formed [29,32,44,45,48,53,54,55].

The progression through diapause phases in each species, population, genotype, or individual depends on physiological processes that remain largely insufficiently understood. Environmental factors substantially modify their expression. These phases should therefore be interpreted within an ecophysiological framework, in which internal regulatory programs interact with external cues.

Several major pathways of physiological regulation can be distinguished through which alternative developmental trajectories are formed within a population. These include endocrine regulation, energetic or metabolic regulation, trophic regulation, and additional regulatory mechanisms.

Endocrine regulation of diapause in insects is generally associated with changes in the activity of the neuroendocrine system. These changes disrupt the normal hormonal balance that otherwise supports the continuation of development. Depending on the developmental stage, neuropeptides, ecdysteroids, and juvenile hormone may play key roles in this process. In general, diapause is accompanied by a reduction or cessation of the production of hormones that stimulate growth, molting, metamorphosis, or reproductive development. By contrast, diapause termination is associated with the restoration of endocrine activity and the return of the organism to direct development. At the larval, pupal, or adult stage, diapause may be controlled by prothoracicotropic hormone, ecdysteroids, and juvenile hormones. Thus, the hormonal system acts as a central link through which external environmental cues are translated into developmental arrest and maintenance of the diapause state [53].

The mechanisms of hormonal regulation of diapause differ among species. In *B. mori*, the role of diapause hormone (DH) as a specialized inducer of diapause has been well demonstrated. In other insects, the ecdysteroid system and its receptors more often play the leading role [53]. In general, several key hormones may be involved in diapause regulation. However, the specific set of hormones depends on the species and on the developmental stage at which diapause occurs [53].

In the review by Gill et al. (2017) [68], within the framework of the hormonal theory of diapause, suppression of neurosecretory cell activity is described as one of the central physiological mechanisms of both obligate and facultative diapause. As a result, the timely production of hormonal signals required for the continuation of direct development does not occur.

For representatives of the genus *Dendrolimus*, this general scheme is also supported by experimental evidence. R.-D. Han et al. (2008) [66] showed that diapausing and non-diapausing larvae of *D. tabulaeformis* differ in the ultrastructure of the corpora allata and prothoracic glands. In non-diapausing larvae, these organs showed signs of higher cellular and secretory activity. In diapausing larvae, by contrast, the observed morphological state was interpreted as functionally less active [66]. Although direct hormone measurements were not performed in that study, the data obtained suggest that diapause in this species is accompanied by reduced activity of the endocrine systems regulating molting and developmental rate. This is consistent with the general view that hormonal reorganization plays a central role in the realization of the diapause state in insects.

Thus, endocrine regulation of diapause can switch the organism from a direct-development trajectory to a delayed-development trajectory through the action of external cues on neuroendocrine centers. However, the hormonal configuration of this switch depends on the species and developmental stage, and its contribution to polymorphism in ontogenetic duration in *Dendrolimus* remains poorly understood.

Diapause may be initiated not only through endocrine mechanisms but also through metabolic reorganization. Such reorganization may include changes in carbohydrate metabolism and the accumulation of specific metabolites.

Metabolic reorganization is one of the key characteristics of diapause. During the pre-diapause period, insects accumulate nutritional reserves. Then, during the diapause initiation phase, direct development ceases. This transition is usually accompanied by regulated metabolic depression. During diapause maintenance, the metabolic rate remains relatively low and stable. This state allows the organism to use accumulated energy reserves economically over a prolonged period of dormancy. Therefore, before entering diapause, the organism must accumulate sufficient nutrient reserves. These reserves are required to meet energetic demands throughout the entire dormant phase and to preserve resources needed for the completion of development after diapause termination [68].

Many diapausing insects differ physiologically from their non-diapausing counterparts of the same species [52]. In diapausing caterpillars, feeding and energy metabolism are slowed down but do not cease completely [44].

In experiments on *D. tabulaeformis*, R.-D. Han et al. [66] showed that diapausing larvae were characterized by increased levels of lipids, trehalose, proteins, and amino acids. At the same time, they showed reduced levels of free fatty acids, water content, and respiration intensity. This combination of traits indicates the accumulation of energy reserves, general suppression of metabolic activity, and the formation of cryoprotective mechanisms [66]. The authors also note that previous studies showed higher cold tolerance in diapausing larvae of the pine moth *D. tabulaeformis* than in non-diapausing larvae [66]. This is consistent with the broader literature data linking diapause with increased resistance to low temperatures [51,96].

Pullin et al. [97] suggested that metabolic depression during diapause may directly contribute to increased cold tolerance. At low temperatures, the high metabolic demands of tissues in non-diapausing insects or in individuals at an early stage of diapause may be difficult to meet. This is because low temperatures inhibit enzyme activity. Such a mismatch between metabolic demands and the capacity of enzymatic systems may partly explain cold-induced mortality in insects whose metabolic rate is not reduced through diapause [66].

This suggests that the physiological basis of polymorphism may involve alternative metabolic and hormonal programs. These programs determine not only entry into diapause but also the depth of metabolic depression. Consequently, they may affect the potential duration of developmental delay and the overall duration of the life cycle [29,66,73].

Trophic factors play an important role in diapause regulation. These include food quality and quantity, as well as the mode of its metabolic utilization. Gill et al. discuss the “food mobilization hypothesis”. According to this hypothesis, the transition to further development depends not only on the accumulation of nutrients. It also depends on the ability of the organism to convert stored resources into a metabolically available form. In non-diapausing organisms, once a certain amount of food has been accumulated and metabolically processed, a hormonal cascade is initiated that supports the continuation of development. In diapausing individuals, the situation is different. Nutrients deposited in the fat body or yolk temporarily remain physiologically “inaccessible”. Their utilization becomes possible only after the action of specific external stimuli, such as low temperature [68].

During facultative summer diapause in *D. superans sibiricus*, a pronounced decrease in trophic activity is observed. This decrease includes reduced food consumption, reduced assimilation, and lower efficiency of food utilization for growth. These changes are accompanied by a sharp decline in the relative growth rate of larvae and by a redistribution of energy expenditure toward the maintenance of vital functions. In diapausing larvae, utilization of ingested food decreases by 20%. In addition, the efficiency of conversion of ingested and assimilated food into larval biomass is reduced by half compared with the control. As a result, the relative growth rate of diapausing larvae decreases sharply, reaching 0.009 mg/day compared with 0.073 mg/day in the control [44].

Across *Dendrolimus*, the available physiological evidence is uneven but still reveals an important contrast between winter diapause and facultative summer diapause. Winter diapause is the better-characterized state and, at the genus level, corresponds to a classical overwintering survival program. In *D. tabulaeformis*, this program includes reduced functional activity of the corpora allata and prothoracic glands, accumulation of lipids, trehalose, proteins, and amino acids, reduced respiration, lower water content, and increased cold tolerance, especially after cold acclimation [29,32,66]. Thus, winter diapause combines endocrine downregulation, metabolic depression, reserve accumulation, and cold-hardiness enhancement. By contrast, facultative summer diapause in the Siberian moth is physiologically documented mainly through trophic and growth-related traits: larvae show reduced food consumption, assimilation, and conversion efficiency, together with a sharp decline in growth rate [44]. This pattern suggests a warm-season program of growth suppression and maintenance rather than the cold-survival syndrome typical of winter diapause. Direct evidence for endocrine shutdown, cryoprotectant accumulation, dehydration, or a distinct heat- or desiccation-resistance phenotype during facultative summer diapause in *D. sibiricus* is still lacking. At the same time, transcriptomic differences between larvae with shorter and prolonged development indicate the involvement of *JH/Met-, FOXO*-, and ecdysteroid-related regulation in prolonged developmental trajectories [64], suggesting that facultative summer diapause is hormonally regulated, but not yet demonstrably through the same physiological configuration as winter diapause. Therefore, winter diapause in *Dendrolimus* should be interpreted as a relatively conserved overwintering physiological syndrome. Facultative summer diapause in the Siberian moth is better regarded as distinct and still insufficiently resolved. It is associated primarily with trophic suppression, slowed biomass accumulation, and redistribution of individuals between fast and delayed ontogenetic trajectories [29,32,44,53,54,64,66,68,73].

Maternal effects represent an additional level of diapause regulation in insects. In this case, the diapause fate of the offspring is determined by the physiological state of the mother. Such effects have been shown to extend not only to embryonic diapause but also to diapause capacity in larvae or pupae of the next generation [53]. Although the mechanisms of this transgenerational transmission remain insufficiently understood, maternal effects can be regarded as an important regulatory pathway. Potentially, this pathway may contribute to the formation of intrapopulation variability in developmental timing.

From a physiological perspective, polymorphism in ontogenetic duration in *Dendrolimus* results from interindividual variability in the neuroendocrine, metabolic, and trophic regulation of seasonal developmental arrest [44,53,54,66,68,73]. External cues, primarily photoperiod and temperature, act through internal physiological systems, but the response to these cues may differ among individuals.

At the intrapopulation level, such regulation may lead to the splitting of individuals within a single population into alternative developmental trajectories. These trajectories differ in growth rate, number of overwintering events, and total life-cycle duration [44,45,66]. At the interspecific and spatial–geographical levels, similar physiological mechanisms may be expressed as differences among species and local populations in typical ontogenetic duration, diapause intensity, and the capacity for prolonged development [29,32,45,48,54,55].

## 7. Molecular Genetic Regulation of Polymorphism in Ontogenetic Duration

From the perspective of genetic mechanisms, polymorphism in ontogenetic duration in insects should not be reduced to the presence of separate genes determining a two- or three-year developmental cycle. In most cases, this polymorphism can be regarded as a polygenic threshold trait. In this context, what is inherited is not a fixed developmental duration but rather a set of parameters of the seasonal switch. These parameters include the threshold for diapause induction, diapause intensity, diapause stability, and the rate of diapause termination, all of which are controlled by multiple genes. The expression of the trait, that is, direct development or diapause, depends on reaching critical values of photoperiod and temperature. Therefore, different genotypes within the same population may respond differently under similar environmental conditions. As a result, intrapopulation polymorphism is formed.

This logic is supported by studies on the invasive stink bug *Halyomorpha halys*. In this species, F1 hybrids showed intermediate values of developmental rate and the proportion of diapausing individuals between the parental forms. This indicates an additive polygenic pattern of inheritance for these traits [98]. Similar data have been obtained for *Chilo partellus*, in which diapause induction and duration are controlled by multiple loci with a threshold effect [99], and for *Ophraella communa*, which is characterized by high heritability of the photoperiodic response [100].

Additional evidence supporting the polygenic nature of diapause comes from studies on *Choristoneura fumiferana*. When diapausing and non-diapausing lines derived from the same population were compared, neither large chromosomal rearrangements nor evidence for a single major-effect locus were detected. Instead, differences between the lines were manifested primarily at the level of transcriptomic regulation. In the diapausing line, pronounced changes were observed in the expression of genes associated with glycolysis, cellular catabolism, signal transduction, and the processing of environmental cues. Moreover, divergence in expression profiles began as early as the first larval instar [78].

A recent study on *Bombyx mori* showed that diapause variability in *Lepidoptera* may also be determined by a specific causal locus associated with the circadian system. The authors identified the N-terminal region of the CYCLE protein as a key factor underlying differences in embryonic diapause. In the silkworm, this gene was found to generate several isoforms through alternative splicing. Disruption of one of these isoforms is associated with the non-diapausing phenotype of polyvoltine lines. Other isoforms apparently retain functions in the regulation of circadian rhythms.

These data indicate that the genetic determinants of diapause may be associated not only with allelic differences. They may also be related to the functional specialization of isoforms of pleiotropic regulatory genes. Such specialization may provide a basis for the evolution of differences in life-cycle duration without disrupting essential basic functions [101].

In representatives of the genus *Dendrolimus*, the genetic basis of polymorphism in ontogenetic duration is apparently realized through a set of inherited mechanisms of seasonal timing. These mechanisms determine individual sensitivity to photoperiodic cues and the probability of transition to diapause.

Experimental studies have shown that, in *D. punctatus*, photoperiod regulates diapause induction [24]. In *D. tabulaeformis*, mechanisms of the photoperiodic clock have been investigated [29]. These data indicate the existence of a genetically determined system of seasonal regulation of development in representatives of the genus *Dendrolimus*.

Additional support for the involvement of the sensory component in this regulation comes from stage-specific and photoperiod-dependent differences in the expression of opsin genes identified in *D. punctatus* [102].

From a genetic perspective, polymorphism in ontogenetic duration in *Dendrolimus* should be regarded as the result of variability in the functioning of the photoperiodic sensory system and associated regulatory cascades. However, the causal role of individual genes in the formation of one-year, two-year, or multi-year developmental trajectories in *Dendrolimus* remains insufficiently studied. Nevertheless, the first tissue-resolved transcriptomic dataset for *D. sibiricus* already permits a pathway-level interpretation of candidate molecular regulators of prolonged development [64].

Stage-specific gene expression apparently makes a substantial contribution to the regulation of ontogenesis in representatives of the genus *Dendrolimus*. In *D. punctatus*, different ontogenetic stages have been shown to be characterized by distinct gene expression profiles. At the egg stage, activation of genes associated with embryogenesis, the cell cycle, and protein synthesis is most typical. At the larval stage, genes involved in digestion and energy metabolism are predominantly activated. At the pupal stage, genes associated with proteolysis, autophagy, and tissue remodeling become particularly important. At the adult stage, genes related to reproduction, olfaction, flight, and metabolism are activated. These data indicate that the rate and trajectory of ontogenesis are not only determined by external factors. They also depend on an internal developmental program that is implemented through the sequential switching of different genetic and transcriptional networks at different stages of the life cycle [103].

Akhanaev et al. [64] presented the first comprehensive transcriptomic study of ontogenetic variability in *D. sibiricus* with different life cycles. Larvae of *D. sibiricus* with one-year and two-year life cycles were shown to have distinct transcriptomic profiles. Moreover, life-cycle type was the main factor structuring gene expression. These differences were predominantly tissue-specific. They were especially pronounced in the fat body and the gut-associated tissue set. Functional enrichment of differentially expressed genes (DEGs) indicates the remodeling of energetic, metabolic, biosynthetic, and structural programs during prolonged development. In larvae following the two-year developmental trajectory, that is, a trajectory with two overwintering events that spans three calendar years, changes were also detected in the expression of components of JH/Met signaling, *FOXO*-associated pathways, JH-binding proteins, P450-related genes, and genes containing ecdysteroid kinase-like domains. These data indicate the involvement of hormonal and stress-associated molecular regulation in the maintenance of prolonged larval development [64].

Akhanaev et al. [64] presented the first tissue-resolved transcriptomic analysis directly comparing *D. sibiricus* larvae expressing the one-year and two-year life-cycle trajectories. The major signal in the dataset was life-cycle type rather than sex, and the strongest sex × life-cycle interaction was confined to the gut-associated tissue set, which also included gonads and Malpighian tubules [64]. The two-year trajectory was not associated with a single diagnostic gene, but with broad and strongly tissue-specific reprogramming. The fat body showed the largest number of DEGs and a mixed signature of upregulated mitochondrial electron transport, ATP synthesis, contractile and proteasome-related functions together with downregulation of ribosome biogenesis, RNA processing, and rRNA processing. The male gut was dominated by suppression of digestive, cilium-related, and anabolic programs, whereas the female gut retained a more biosynthetic profile. In the head tissue set, genes related to translation, vesicle transport, dopamine metabolism, and muscle-associated processes were differentially expressed [64]. Taken together, these results indicate that prolonged development in *D. sibiricus* involves coordinated metabolic repartitioning among tissues rather than a simple uniform slowdown of the whole organism.

At the pathway and gene-family level, the principal candidates identified so far in *D. sibiricus* include juvenile-hormone signaling components centered on Methoprene-tolerant (Met). Downstream or associated modules include *PI3K/AKT, STAT3, RAP1/RAC1, PTK2,* and *PTPN11*. Other candidates include *FOXO*-associated regulators, hemolymph juvenile hormone-binding proteins, cytochrome P450 family genes, and genes containing ecdysteroid kinase-like domains [64]. Together with the enrichment of catabolic-remodeling, stress-tolerance, detoxification, proteostasis, and oxidative-phosphorylation modules, this pattern supports the interpretation that the two-year trajectory reflects molecular features of facultative summer diapause, including growth suppression and endocrine and metabolic reorganization [64]. However, important gaps remain. The available evidence is based on poly(A)-selected larval RNA-seq and therefore does not address regulatory non-coding RNAs, including miRNAs and lncRNAs, alternative splicing, allele-specific expression, or transposable-element activity. No data are yet available for DNA methylation, histone modifications, chromatin accessibility, or other epigenetic mechanisms that could mediate environmentally induced or transgenerational stabilization of fast versus delayed trajectories. In addition, transcriptomic signatures have not yet been linked to direct measurements of juvenile hormone or ecdysteroid titres, nor functionally tested by RNAi, CRISPR/Cas, or endocrine manipulation. Thus, for *D. sibiricus*, the current molecular-genetic model should be regarded as a pathway-level hypothesis that identifies plausible candidate regulators of prolonged development, while the non-coding and epigenetic layers of regulation remain essentially unexplored.

## 8. Integration of Ontogenetic Polymorphism into Outbreak Prediction Models

Existing prediction systems for *Dendrolimus* pests combine meteorological and hydrothermal variables, accumulated heat, precipitation and drought indices, host-stand composition, site quality, topography, direct population indicators, pheromone-monitoring data, and remote-sensing metrics of stand condition [17,20,35,37,40,41,42,43,63]. Models for other *Dendrolimus* species, including *D. superans*, *D. pini*, *D. punctatus*, and *D. spectabilis* [19,22,26,28], are useful here only as a methodological analogy. They identify environmental predictors that can be tested for the Siberian moth. However, they do not constitute evidence that facultative summer diapause or diapause-driven fast/delayed splitting occurs in those species.

For Siberian moth these predictors can be reframed as possible drivers of a hidden developmental-state structure rather than as predictors of abundance alone. Several factors may influence whether larvae remain on a fast trajectory or shift toward delayed development. These factors include heat accumulation, warm dry periods, drought-induced food limitation, host-plant quality, larval density, overwintering conditions, and larval physiological state [4,12,44,51,53,54,59,60,61,62,73]. In such a model, the key output would not be only expected population density but also the fast/delayed cohort ratio, the probability of facultative summer diapause, and the expected overlap of adult emergence.

This link is operationally important because calendar age may not match physiological age. Monitoring based on a single expected larval age class can underestimate the delayed fraction, and chemical or biological treatments timed for one vulnerable cohort may leave larvae that are out of phase with that window. Baranchikov et al. [46] and subsequent summaries [4,12,59] indicate that, during outbreaks, short- and long-cycle fractions can contribute adults to the same flight season; this temporal convergence may sharply increase apparent abundance, reduce the predictability of flight peaks, and make one-window control unreliable.

## 9. Discussion

The literature reviewed here suggests that at least part of the apparent inconsistency among studies on ontogenetic-duration variation in *Dendrolimus* reflects differences in taxonomic interpretation, environmental context, and study design rather than direct biological contradiction. This problem is most evident in the Siberian moth, where developmental and outbreak data have been published under the names *D. sibiricus, D. superans sibiricus*, or *D. superans*, depending on the taxonomic concept adopted by the authors. As a result, records from Siberia, the Russian Far East, northeastern China, Sakhalin, and Japan are not always fully comparable. In addition, populations compared across studies often differ in thermal regime, growing-season length, host composition, drought exposure, outbreak phase, and local trophic conditions. Therefore, differences in reported life-cycle duration, diapause expression, or the frequency of prolonged development should not automatically be interpreted as evidence of mutually exclusive mechanisms. In many cases, they more likely reflect the fact that different studies captured different positions along a continuum of seasonal constraints and developmental thresholds [1,3,4,5,6,7,8,9,10,12,18,45,46,53,59,60,61,62,63,64].

Current phenological models for *Dendrolimus* are useful, but they remain uneven in explanatory power. Degree-day, climatic-suitability, and weather-based outbreak models successfully describe broad spatial patterns, regional voltinism shifts, and climate-related changes in outbreak risk. This is well illustrated by models and forecasting approaches developed for *D. pini*, *D. spectabilis*, *D. superans*, and *D. sibiricus* under changing thermal conditions [12,17,19,20,22,26,28,35,37,40,41,42,43,57,63]. However, these models generally treat development as if populations followed a single dominant trajectory or a cohort-average schedule. Such an approach is adequate when the main question is climatic suitability or mean developmental timing, but it becomes insufficient when a population contains a hidden mixture of fast- and delayed-developing individuals. For the Siberian moth in particular, the key limitation of current models is that facultative summer diapause is still not represented as an explicit state variable. Consequently, existing models may correctly predict favorable years for rapid development or outbreak escalation yet still fail to estimate the proportion of larvae shifted to delayed trajectories, the overlap of adults from different cohorts, or the resulting distortion of monitoring signals. For this reason, future phenological models for *D. sibiricus* should move from simple cohort-means toward state-structured or probabilistic frameworks in which the fast/delayed split is itself modelled as a biologically meaningful outcome rather than treated as unexplained noise [4,12,17,35,37,44,45,46,53,63,64].

The discrepancy between field and laboratory results can also be reconciled within this framework. Field observations and long-term phenological interpretations have repeatedly linked prolonged development in the Siberian moth with trophic deterioration, starvation, abnormal temperatures, drought-related stress, and outbreak-related changes in stand condition [4,12,44,45,59,60,61,62,63]. By contrast, the recent laboratory study of Akhanaev et al. [64] showed that larval density, food deprivation, and host-plant species, when tested separately under controlled conditions, did not independently induce switching to the prolonged developmental trajectory, although they clearly affected mortality, stress, and body-mass gain. These results are not contradictory. Rather, they indicate that facultative summer diapause in *D. sibiricus* is unlikely to be triggered by any single isolated factor. Field conditions expose larvae to fluctuating temperature regimes, changing food quality, repeated stress, ecological history, and possibly maternal or transgenerational effects, whereas laboratory experiments usually test individual factors separately and under constant or simplified conditions. In other words, field studies primarily capture the realized developmental outcome of a complex ecological sequence, while laboratory studies test only a limited subset of candidate cues. The negative laboratory result therefore argues against a simple single-factor induction model, not against the biological reality of facultative summer diapause itself [44,45,53,54,59,60,61,62,63,64,68,91].

Taken together, the most parsimonious interpretation is that ontogenetic-duration polymorphism in *Dendrolimus* is generated by threshold-dependent regulation acting in an ecologically heterogeneous background, but that the mechanistic architecture of this threshold differs among taxa and among diapause types. In the genus as a whole, winter diapause can be treated as the better-resolved and more conserved seasonal module. In the Siberian moth, however, delayed development appears to depend on a more weakly resolved interaction among climatic conditions, trophic state, developmental history, and endocrine or molecular timing mechanisms. This interpretation helps reconcile why the existence of delayed trajectories is well supported in natural populations, whereas the exact induction mechanism remains experimentally unresolved. This interpretation also has direct practical significance for forecasting and pest management. The hidden delayed fraction should be treated as an intrinsic component of population structure, not as an anomaly. It can alter emergence synchrony, outbreak tempo, and the reliability of control measures timed to a single expected cohort [4,12,20,35,36,37,38,39,44,45,46,53,63,64,66].

## 10. Conclusions

Polymorphism in ontogenetic duration in *Dendrolimus* should be regarded as a multilevel phenomenon rather than as simple variation in voltinism. It is expressed at intrapopulation, interspecific, and spatial–geographical levels, but the strongest evidence for natural within-population splitting is currently available for the Siberian moth, in which fast and delayed developmental trajectories coexist and appear to be associated with facultative summer diapause.

The available evidence indicates that this hidden developmental heterogeneity is biologically important but still insufficiently incorporated into phenological forecasting. Explicit consideration of the delayed fraction, rather than reliance on mean developmental timing alone, should improve outbreak prediction, risk assessment, and the timing of monitoring and control measures for *Dendrolimus* pests.

Future work should therefore focus on three linked directions. First, long-term field monitoring is needed to estimate the proportion of fast- and delayed-developing larvae across populations, outbreak phases, and climatic gradients. Second, experiments should identify the combined environmental, physiological, and molecular cues that induce or prevent facultative summer diapause. Third, developmental-state structure should be incorporated into phenological and outbreak-risk models, including projections under climate change and cross-regional comparisons.

## Figures and Tables

**Table 1:** Comparative evidence for different levels of ontogenetic-duration polymorphism in selected *Dendrolimus* taxa.

Species/Taxon	Main Level of Polymorphism	Possible Triggers or Conditions	Developmental Outcome	Evidence Status
*D. sibiricus*/*D. superans sibiricus*	Intrapopulation and spatial–geographical levels [4,6,8,9,18,44,45].	Heat accumulation, drought, population density, food quality, overwintering conditions, and larval physiological state [12,45,59,60,61,62,63,64].	Fast and delayed developmental trajectories; two- and three-calendar-year cycles; in extreme cases, one-year or four- to five-year variants [4,6,8,9,18,44,45,46].	Strongest evidence for natural intrapopulation splitting of developmental trajectories [46,59,60,61,62,63,64].
*D. punctatus*	Intrapopulation splitting near threshold conditions; population differences in voltinism [24,58].	Critical photoperiod, temperature, and host-needle quality or damage [24,58].	Partial diapause induction: some larvae continue development, whereas others enter diapause [58].	Experimental evidence for partial splitting, but not for facultative summer diapause [58].
*D. pini*	Spatial–geographical level [20,38].	Thermal regime, climatic conditions, position within the range, and photoperiodic regulation of diapause [20,38,60,65].	Variation in life-cycle duration from one to three years [20,38].	Mainly evidence for geographically and thermally structured variability [20,38].
*D. spectabilis*	Spatial–geographical level; changes in voltinism [28,56].	Accumulated effective temperatures, climate warming, and latitudinal or altitudinal gradients [28,56].	Shift from univoltine to bivoltine development under some conditions [56].	Modeling and field evidence for changes in voltinism [28,56].
*D. tabulaeformis*	Physiological and diapause-related variability [29,32,66].	Photoperiod, temperature, and cold acclimation [29,32,66].	Diapausing and non-diapausing states; differences in cold tolerance, metabolism, and endocrine activity [29,32,66].	Strong physiological evidence, but less direct evidence for natural population-level polymorphism [29,32,66].
*D. superans* s. str., *D. houi*, and *D. kikuchii*	Poorly resolved; mainly ecological, biogeographical, and phylogenetic context [14,15,16,17,18,19,33,34,57].	Distribution, host plants, and climatic suitability of habitats [14,15,16,17,18,19,33,34,57].	Insufficient evidence for confidently describing alternative developmental trajectories [14,15,16,17,18,19,33,34,57].	Useful for comparative context, but not for inferring diapause-driven intrapopulation polymorphism [14,15,16,17,18,19,33,34,57].

**Table 2:** Evidence on facultative summer diapause in the Siberian moth.

Study	Study Type and Conditions/Factors Considered	Direct Observation or Result	Interpretation in Relation to Facultative Summer Diapause	Evidence Robustness
Rozhkov [6]	Classical field and phenological synthesis across different parts of the species range; life-cycle duration and the number of overwintering events were considered.	The life cycle of the Siberian moth may vary in duration and may include different numbers of overwintering events.	Provides the phenological background for recognizing alternative developmental trajectories in this species.	Historical phenological evidence; important as a baseline, but not a direct experimental test of facultative summer diapause induction.
Baranchikov and Kirichenko [44]	Study of feeding and growth of larvae during facultative summer diapause; trophic activity and growth parameters were assessed in diapausing larvae.	Larvae in facultative summer diapause showed reduced food consumption, reduced assimilation, lower efficiency of food conversion, and sharply decreased growth rate.	Demonstrates that facultative summer diapause is associated with a distinct trophic and physiological state.	Direct evidence for the physiological expression of facultative summer diapause; limited evidence for the cues inducing this state.
Kirichenko et al. [45]	Laboratory rearing on potted host plants under long-day conditions (L:D 18:6); larval performance and life-cycle completion were assessed.	Some larvae did not complete development by the end of the experiment despite long-day conditions.	Suggests that developmental delay may occur outside direct dependence on classical short-day photoperiodic induction.	Experimental evidence for developmental delay under controlled long-day conditions; indirect evidence for facultative summer diapause.
Baranchikov et al. [59]	Field observations in a defoliated larch forest; population age structure and trophic conditions were considered.	Changes in population age structure were observed under conditions of defoliation and trophic deterioration.	Supports the hypothesis that starvation or deteriorating food conditions may be associated with facultative summer diapause and delayed development.	Field-based evidence; ecologically relevant, but not a controlled experimental test.
Geispits [60]	Study of photoperiodic and temperature reactions determining seasonal development.	Photoperiod and temperature were identified as important regulators of seasonal development in pine moths, including the Siberian moth.	Provides historical support for the role of environmental cues in regulating seasonal development.	Historical and comparative evidence; relevant to environmental regulation, but not a direct test of facultative summer diapause in *D. sibiricus.*
Konikov et al. [61]	Historical experimental and observational study of adaptation to environmental conditions; factors affecting population level were considered.	Environmental conditions were discussed as factors influencing development and population dynamics of the Siberian moth.	Supports the general hypothesis that developmental trajectories may depend on external environmental context.	Historical evidence; useful for hypothesis generation, but experimental detail is limited by modern standards.
Konikov et al. [62]	Study focused on the role of diapause in the alimentary adaptation of Siberian moth larvae.	Diapause was considered in relation to larval feeding conditions and alimentary adaptation.	Supports the hypothesis that trophic conditions may be involved in the regulation of facultative summer diapause.	Historical evidence directly relevant to the trophic interpretation of diapause, but with limited experimental resolution.
Akhanaev et al. [64]	Laboratory testing of three factors separately: larval density, food deprivation, and host-plant species.	High larval density did not independently trigger prolonged development. Starvation induced stress and mortality but did not prolong development. Host plant affected mortality and body-mass gain, but surviving larvae pupated successfully.	Indicates that the switch to a prolonged developmental trajectory is unlikely to be induced by any of these factors alone.	Direct experimental evidence against single-factor induction; robust for the tested factors, but does not exclude multifactorial, sequential, ecological-history, or transgenerational effects.
Demidko et al. [63]	Weather-data-based outbreak prediction model; meteorological predictors several years before outbreak onset were analyzed.	Weather conditions preceding outbreaks were interpreted as favoring faster larval development and the avoidance of facultative summer diapause.	Proposes avoidance of facultative summer diapause under favorable weather conditions as a mechanism contributing to accelerated development and outbreak formation.	Model-based inference; important for outbreak prediction, but not a direct experimental test of facultative summer diapause induction.

## Data Availability

No new data were created or analyzed in this study. Data sharing is not applicable to this article.
